# Gene profiling of human VEGF signaling pathways in human endothelial and retinal pigment epithelial cells after anti VEGF treatment

**DOI:** 10.1186/1756-0500-7-617

**Published:** 2014-09-08

**Authors:** Shani Golan, Michal Entin-Meer, Yonathan Semo, Sofia Maysel-Auslender, Daphna Mezad-Koursh, Gad Keren, Anat Loewenstein, Adiel Barak

**Affiliations:** Departments of Ophthalmology, Tel-Aviv Sourasky Medical Center, Sackler Faculty of Medicine, Tel Aviv University, Tel-Aviv, 64239 Israel; Departments of Cardiology, Tel-Aviv Sourasky Medical Center, Sackler Faculty of Medicine, Tel Aviv University, Tel-Aviv, Israel

**Keywords:** Bevacizumab, Ranibizumab, Gene expressions, VEGF, PCR

## Abstract

**Background:**

Ranibizumab (Lucentis®) is a Fab-antibody fragment developed from Bevacizumab, a full-length anti-VEGF antibody. Both compounds are used for treating age-related macular degeneration (AMD). The influence of bevacizumab and ranibizumab on genes involved in signal transduction and cell signaling downstream of VEGF were compared in order to detect possible differences in their mode of action, which are not related to their Fab-antibody fragments.

**Methods:**

Human umbilical vein cell lines (EA.hy926) and retinal pigment epithelial cells (ARP-19) were exposed to oxidative stress. The cells were treated with therapeutic concentrations of bevacizumab (0.25 mg/mL) and ranibizumab (125 mg/mL) for 24 hours prior to all experiments, and their effects on gene expressions were determined by RT- PCR.

**Results:**

After exposure to bevacizumab, more genes in the endothelial cells were up-regulated (KDR, NFATc2) and down-regulated (Pla2g12a, Rac2, HgdC, PRKCG) compared to non-treated controls. After exposure to ranibizumab, fewer genes were up-regulated (PTGS2) and down-regulated (NOS3) compared to controls. In comparison between drugs, more genes were up-regulated (NFATc2 and KDR) and more were down-regulated (Pla2g12a, Pla2g1b, Ppp3r2, Rac2) by bevacizumab than by ranibizumab. In RPE cells, NOS3 and PGF were up-regulated and Pla2g12b was down-regulated after exposure to ranibizumab, while PIK3CG was up-regulated and FIGF was down-regulated after exposure to bevacizumab, but the differences in gene expression were minor between drugs (PIK3CGand PGF were down-regulated more by ranibizumab than by bevacizumab).

**Conclusions:**

The different gene expressions after exposure to ranibizumab and bevacizumab in endothelial and RPE cells may indicate a somewhat different biological activity of the two compounds.

## Background

Age-related macular degeneration (AMD) is the leading cause for legal blindness among the elderly in the industrialized world. Pathological angiogenesis is the underlying cause of the exudative form of AMD.

VEGF-A is a potent endothelial cell mitogen, and recent evidence indicates that it acts as an autocrine growth and survival factor in VEGF-A-producing cells [1, 2]. Several studies suggest that it is a major mediator of angiogenesis and vascular leakage in exudative AMD [3, 4]. Inhibition of VEGF-A activity has been a central theme in many therapies under investigation. Several inhibitors have been developed and are now used clinically.

In 2006, ranibizumab (Lucentis; Genentech, Inc., San Francisco, CA, USA), an antibody fragment developed against all fragments of VEGF, was approved by the FDA for treating AMD following the MARINA [5] and the ANCHOR [6] studies, which showed that ranibizumab had a stabilizing effect in 90% of the patients and a beneficial effect in 30%.

Ranibizumab is highly effective but considerably expensive. In contrast, bevacizumab, a full-length anti-VEGF antibody approved for use in colon cancer [7], costs much less than ranibizumab.

Ocular use of bevacizumab is currently not FDA approved; nevertheless, it is widely used for treating AMD [8–11]. Bevacizumab has been described in case reports [8–10], retrospective studies [10, 11], and controlled prospective studies (CATT and IVAN trials) as effective and well tolerated. To date, only limited experimental data and studies comparing the effects of bevacizumab and ranibizumab have been published. The comparative trials CATT and IVAN evaluated the therapeutic efficacy of bevacizumab compared with ranibizumab, concluding that the former was not inferior to the latter [12, 13].

Despite the fact that ranibizumab and bevacizumab share the same Fab-fragment blockage properties, they are actually different molecules. Bevacizumab is produced in Chinese hamster ovary (CHO) cells from the expression plasmids VID5.ID.LLnspeV.xvegf36HC.LC. The resulting antibody is produced from the G7 clone as a 149-kDa full-length IgG1 antibody composed of two 214-residue light chains and two 453-residue heavy chains. Each light chain is covalently linked to a heavy chain and the two heavy chains are covalently bonded. The resulting bevacizumab antibody contains 93% human amino acid sequence [14, 15].

Ranibizumab was developed in an effort to obtain higher affinity variants of Fab-12 with improved potency and efficacy [16]. Although bevacizumab was derived from Fab-12, ranibizumab was derived from an in vitro CDR mutation and affinity selection from a different humanized anti-VEGF Fab variant, known as MB1.6 [17, 18]. Ranibizumab is produced as a 48 kDa Fab in *Escherichia coli* from the expression plasmid pY0317. The heavy and light chains fold into their native conformation following secretion into the bacteria’s periplasmic space and are covalently linked. The resulting Fab-Y0317 is known as ranibizumab [19, 20].

It has been shown previously that the two molecules act differently and posses' different pathway activities which may be unrelated to their anti-VEGF activities [21–23].

As bevacizumab and ranibizumab differ in their molecular composition and physiologic properties, the present study compared VEGF inhibitors in terms of their effects on genes involved in signal transduction and cell signaling downstream of VEGF.

The genes selected are all genes expressed downstream the VEGF pathway in the cell after receptor dimerization and autophosphorylation.

The expressions of genes directly mediating VEGF signaling were analyzed to detect differences in molecular pathways when both compounds are applied. The chosen model was designed to compare the effects of VEGF inhibitors on specific genes expressed in the angiogenesis/vasculogenesis process in both RPE and endothelial cells.

Cellular damage resulting from oxidative stress in both endothelial and RPE cells plays a causative role in AMD [3]. Oxidative stress-induced RPE cell apoptosis has been proposed as a major pathophysiological mechanism of AMD [3, 22–24]. In particular, RPE cell apoptosis is an important feature of the advanced form of dry AMD [3, 25] Thus, oxidative stress induces VEGF-A expression from the RPE and also RPE death [3, 22], suggesting a role for such stress in both neovascular and advanced dry AMD.

The effects on gene expression were examined using a model of ischemia (12 hours in a hypoxic chamber) to mimic significant stress imposed upon the cells in neovascular AMD in real time.

## Methods

### Cell culture

EA.hy926 cells (a human umbilical vein cell line) were seeded at 100,000/cm [2] in T-75 cm [2] flasks containing DMEM with 15 Mm Hepes buffer, 10% fetal bovine serum, 2 mM L-glutamine solution and 10% pen-strep at 37°C for 1 week. Serum was withdrawn in DMEM + 1% bovine serum albumin for 3 days to make the cells quiescent. ARPE-19 cells were seeded on 1*10 [6]/10 cm plates containing DMEM with 10% fetal bovine serum, 1% L-glutamine solution, and 10% pen-strep at 37°C for 1 week, and serum was withdrawn in DMEM + 1% bovine serum albumin for 3 days to make the cells quiescent.

### Exposure to bevacizumab and ranibizumab

Before all experiments, both cell lines were treated for 12 hours in a hypoxic chamber (exposure to less than 2% oxygen in the chamber). Therapeutic dosages of both bevacizumab and ranibizumab (0.25 mg/mL and 0.125 mg/mL, respectively) were then added to the cell lines. These concentrations were prepared using serial dilutions of the drug in the respective serum-free culture medium. The solution of the drug mixed with media was then directly added to the cells in order to obtain a uniform concentration of drug throughout the well of the tissue culture plate. In addition to bevacizumab and ranibizumab, the cells were also treated with 10 ng/ml hVEGF (PeprotechInc, Rocky Hill, NJ, USA).

### Control groups

All experiments were compared to controls. Controls were cells that had been treated with human VEGF (hVEGF) alone and no bevacizumab or ranibizumab.

### RNA production

After 48 hours of exposure to either ranibizumab or bevacizumab, the total cellular RNA was isolated from the cells by a QiagenRNeasy® Mini Kit (Catalog # 74104) according to the manufacturer's instructions. RNA samples underwent DNase treatment and removal (QiagenRNeasy® Mini Kit, Catalog # 74104). RNA quantification was performed with spectrophotometry (ND-1000; NanoDrop Products, Thermo Fisher Scientific, Wilmington, DE), after which 250 ng of total RNA was analyzed by agarose gel electrophoresis to confirm integrity. The resultant RNA was stored at -80°C.

The RNA was reverse transcribed using the RT [2] First Strand Kit (Qiagen).

### Real-time quantitative (q) RT-polymerase chain reaction (PCR)

A SAB biosciences RT [2] Profiler PCR Array assay (Qiagen) was performed according to the manufacturer's instructions, using syber green [26].

The RT array included five controls for housekeeping genes, one control for genomic DNA and three reverse transcription controls (no RNA loaded).three positive RNA controls were also present. PCR was performed on an ABI Prism 7700 Sequence Detector (©2008 Applied Biosystems. All rights reserved. Applera, Applied Biosystems). The data were analyzed using the web-based software RT2 Profiler PCR Array data analysis tool, (Qiagen). The ΔΔCt method was used for data analysis. Specifically, fold-changes for each gene were calculated as difference in gene expression between bevacizumab exposure and control, and between ranibizumab exposure and control. A positive value indicated gene up-regulation and a negative value indicated gene down-regulation.

### Statistical analysis

Each experiment was independently repeated at least twice (as recommended by the manufacturers guidelines for statistical significance). Genes with greater than 2.5 fold change in expression compared to control were identified as significant (p < 0.05).Only results that showed a relatively high (>30) average threshold cycle of a gene in either the control or the test sample, and a reasonably low (<30) average threshold cycle in the other sample were included. Data that demonstrated the gene’s expression was relatively low in one sample and reasonably detected in the other sample suggested that the actual fold-change value was at least as large as the calculated and reported fold-change result. The results that fitted this formula were graded “A”. Results that were graded “B” indicated that the gene’s average threshold cycle was relatively high (>30), implying that its expression level was relatively low in both the control and test samples, and that the *P* value for the fold-change was relatively high (*P* > 0.05). All experiments were repeated at least twice. The list of genes examined is summarized in Table 1.

**Table 1:** **Positions of genes and genes symbol**

Position	Symbol	B11	MAPK12
A01	AKT1	B12	MAPK13
A02	AKT2	C01	MAPK14
A03	AKT3	C02	MAPK3
A04	ARNT	C03	MAPKAPK2
A05	BAD	C04	MAPKAPK3
A06	CASP9	C05	NFAT5
A07	CAV1	C06	NFATC1
A08	CDC42	C07	NFATC2
A09	FIGF	C08	NFATC3
A10	FLT1	C09	NFATC4
A11	FLT4	C10	NOS3
A12	GRB2	C11	NRAS
B01	HIF1A	C12	NRP1
B02	HRAS	D01	NRP2
B03	HSP90AA1	D02	PDGFC
B04	HSPB1	D03	PGF
B05	KDR	D04	PIK3CA
B06	KRAS	D05	PIK3CB
B07	MAP2K1	D06	PIK3CD
B08	MAP2K2	D07	PIK3CG
B09	MAPK1	D08	PIK3R1
B10	MAPK11	D09	PIK3R2
D10	PIK3R3	F10	PRKCG
D11	PIK3R5	F11	PTGS2
D12	PLA2G10	F12	PTK2
E01	PLA2G12A	G01	PXN
E02	PLA2G12B	G02	RAC1
E03	PLA2G1B	G03	RAC2
E04	PLA2G2A	G04	RAF1
E05	PLA2G2D	G05	SH2D2A
E06	PLA2G2E	G06	SHC2
E07	PLA2G2F	G07	SPHK1
E08	PLA2G3	G08	SPHK2
E09	PLA2G4A	G09	SRC
E10	PLA2G4B	G10	VEGFA
E11	PLA2G5	G11	VEGFB
E12	PLA2G6	G12	VEGFC
F01	PLCG1	H01	B2M
F02	PLCG2	H02	HPRT1
F03	PPP3CA	H03	RPL13A
F04	PPP3CB	H04	GAPDH
F05	PPP3CC	H05	ACTB
F06	PPP3R1	H06	HGDC
F07	PPP3R2	H07	RTC
F08	PRKCA	H08	RTC
F09	PRKCB	H09	RTC
H10	PPC		
H11	PPC		
H12	PPC		

## Results

**Figure 1:**
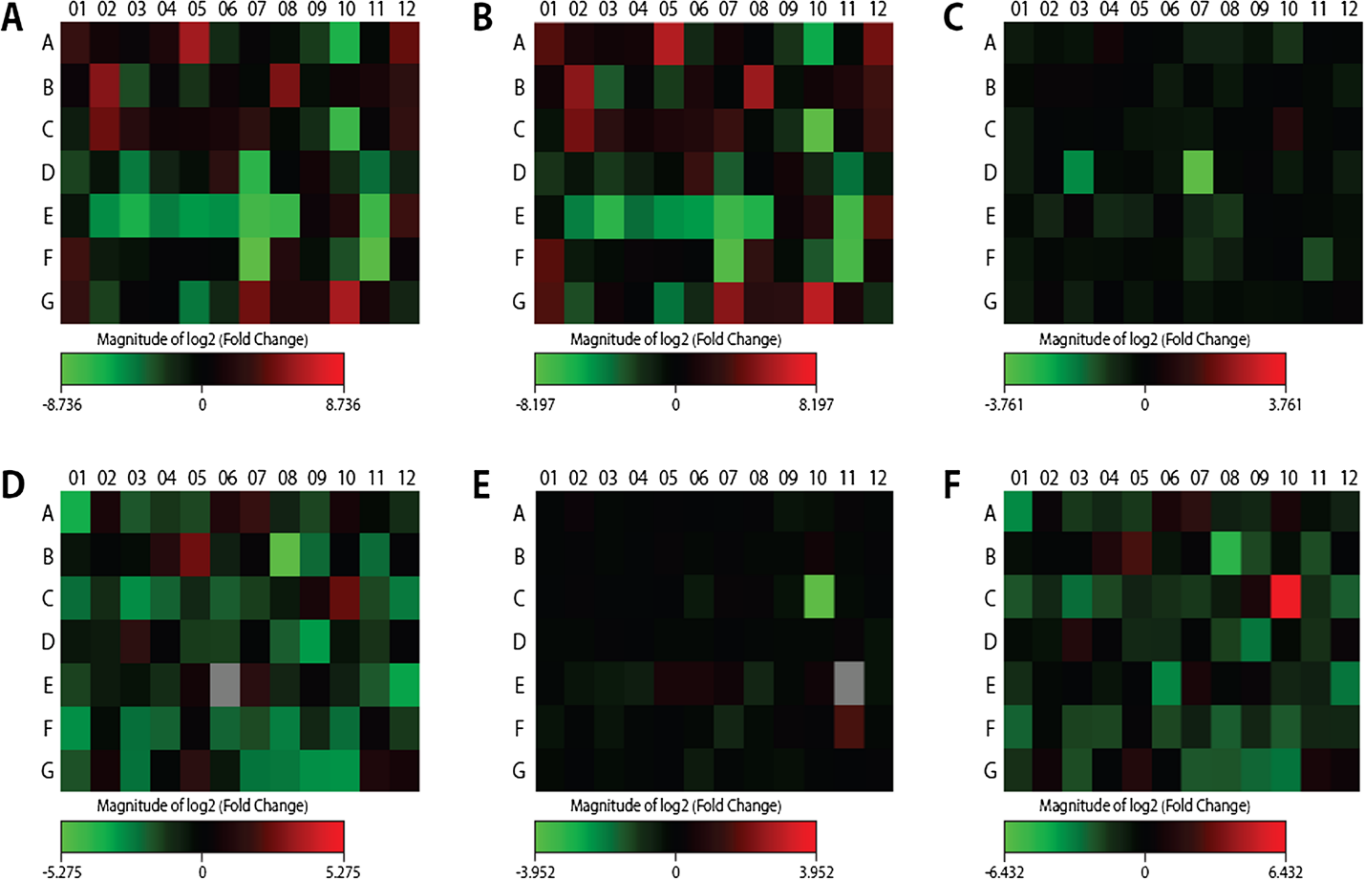
**Heatmap of the altered expression of gens in the different groups compared. A**. Heatmap of the results showing genes with significantly altered transcription levels as a function of exposure to bevacizumab versus non-treated control in RPE cells. **B.** Heatmap of the results showing ranibizumab versus human VEGF treated control in retinal pigment epithelial cells. **C.** Heatmap of the results showing bevacizumab versus ranibizumab in RPE cells. **D.** Heatmap of the results showing bevacizumab versus human VEGF-treated control in endothelial cells. **E.** Heatmap of the results showing ranibizumab versus human VEGFreated control in endothelial cells. **F.** Heatmap of the results showing bevacizumab versus ranibizumab in endothelial cells.

A comparison of the various gene expression patterns of RPE cells after exposure to both anti-VEGF agents and exposure solely to hcVEGF (control) are presented in Tables 2,3 and 4 and Figure 1A-C. Several genes of RPE cells that were treated with bevacizumab were over-expressed compared to controls (MAPK genes, the SPHK gene and the VEGFA gene), while others (the KDR, NOS, PIK3R and PLA2Ggenes) were under-expressed. When the RPE cells were treated with ranibizumab, the NFATC, MAPK, SPHK and VEGFA genes were up-regulated, whereas the KDR and NOS3 genes were under-expressed compared to controls. Only the PGF and PIK3CG genes were down-regulated in the RPE cells that had been treated with bevacizumab compared to when they had been treated with ranibizumab.

**Table 2:** **Fold change of gene expression in retinal pigment epithelial cells exposed to bevacizumab compared to human vascular endothelial growth factor-treatedcontrol**

Gene symbol	Gene name	Bevacizumab/control	Grade
MAPK3	Mitogen-activated protein kinase 3	19.7066	A
NFATC2	nuclear factor of activated T-cells	5.2333	A
NRP1	Neuropilin 1	7.0536	A
SPHK1	Sphingosine kinase 1	22.0982	A
VEGFA	Vascular endothelial growth factor A	61.0411	A
NOS3	Nitric oxide synthase	-170.2534	A
NRP2	Neuropilin 2	-6.2684	A
PIK3R3	Phosphoinositide-3-kinase, regulatory subunit 3 (gamma)	-4.0044	A
PIK3R5	Phosphoinositide-3-kinase, regulatory subunit 5	-14.6532	B
PLA2G2A	Phospholipase A2, group IIA	-21.5369	B
PLA2G2D	Phospholipase A2, group IID	-40.967	B
PLA2G2E	Phospholipase A2, group IIE	-31.6041	B
PLA2G2F	Phospholipase A2, group IIF	-212.0617	B

**Table 3:** **Fold change of gene expression in retinal pigment epithelial cells exposed to ranibizumab compared to human vascular endothelial growth factor-treated control**

Gene symbol	Gene name	Ranibizumab/control	***Grade***
NOS3	Nitric oxide synthase	-293.5515	A
KDR	Kinase insert domain receptor	-5.0041	A
MAPK3	Mitogen-activated protein kinase 3	+20.0674	A
VEGFA	Vascular endothelial growth factor	+73.4215	A
PIK3CG	Phosphoinositide-3-kinase, catalytic, gamma polypeptide	+7.1785	A
SPHK1	Sphingosine kinase 1	+28.9047	A
PLA2G2E	Phospholipase A2, group IIE	-26.829	B
PLA2G2F	Phospholipase A2, group IIF	-123.5516	B
PLA2G2A	Phospholipase A2, group IIA (platelets, synovial fluid)	-12.4228	B
PLA2G1B	Phospholipase A2, group IB	-89.4568	B

**Table 4:** **Fold change of gene expression in retinal pigment epithelial cells exposed to bevacizumab compared to ranibizumab**

Gene symbol	Gene name	Ranibizumab/bevacizumab	***Grade***
Pgf	placental growth factor	-4.2627	A
Pik3cg	phosphoinositide-3-kinase, catalytic, gamma polypeptide	-13.5561	A

Comparisons of the various gene expressions of endothelial cells after exposure to bevacizumab, ranibizumab, and controls are shown in Table 5 and Figure 1D. After exposure to ranibizumab, PTGS2 was up-regulated compared to controls (hVEGF), while NOS3 expressions were down-regulated.

**Table 5:** **Fold change of gene expression in endothelial cells exposed to Bevacizumab or Ranibizumab compared to control**

Gene symbol	Gene name	Bevacizumab/control	***Grade***
KDR	kinase insert domain receptor	+ 6.3173	A
FIGF	C-fos induced growth factor (vascular endothelial growth factor D)	-3.1746	A
PLA2G5	Phospholipase A2, group V	-3.928	B
PRKCG	Protein kinase C gamma	-5.0676	B
Gene symbol	Gene name	Ranibizumab/control	*Grade*
NOS3	nitric oxide synthase 3	-14.8	A

After exposure to bevacizumab, different genes were up- and down-regulated compared to controls (Table 6 and Figure 1E, F). The KDR gene was over-expressed and the FIGF gene was under-expressed. However, in cells treated with ranibizumab, only the NOS3 gene expression was down-regulated compared to controls. Comparison of the two treatment arms revealed that the KDR gene was significantly up-regulated in the cells treated with bevacizumab compared to those treated with ranibizumab, and that the PRKCG gene was down-regulated.

**Table 6:** **Fold change of gene expression in endothelial cells exposed to bevacizumab compared to endothelial cells exposed to ranibizumab**

Gene symbol	Gene name	Bevacizumab/ranibizumab	***Grade***
KDR	Kinase insert domain receptor	+5.547	A
NOS3	Nitric oxide synthase 3 (endothelial cell)	+86.3228	A
NFATC2	Nuclear factor of activated T-cells	-3.3708	A
PLA2G2E	Phospholipase A2, group IIE	-11.1207	B
PRKCG	Protein kinase C, gamma	-5.3751	B

## Discussion

The results of the present study suggest that gene expressions differ after exposure to ranibizumab and bevacizumab in RPE and endothelial cell lines. This finding may indicate that although they both block the VEGF signaling pathway, they probably do so in a somewhat different biological mechanism, or through different transmitters.

RPE cells exposed to either bevacizumab or ranibizumab exhibit different gene expression as compared to controls. Mainly MAPK and VEGFA are over-expressed when treated with either bevacizumab or ranibizumab compared to controls, and the expression of KDR and NOS genes was down-regulated. However, when the two treatment arms were compared (bevacizumab compared to ranibizumab), no major differences in gene expression are noted (besides PGF and PIK3CG). Both gene expressions play a role in the VEGF receptor dimerization and autophosphorylation, and play a crucial role in the VEGFA mitogenic signaling pathway.

In endothelial cells, the situation was somewhat different. No major gene expression was noted when comparing both treatment arms to control; however, when comparing bevacizumab to ranibizumab, major differences in gene expressions were noted (KDR and NOS3 were up-regulated and NFATc2 was down regulated with bevacizumab).NFATc2 is a calcineurin/nuclear factor of activated T cells c2 (NFATc2) pathway and has displayed an anti-apoptotic role in melanoma cells [27].

VEGF is a heparin-binding homodimeric glycoprotein that acts via endothelial-specific receptor tyrosine kinases, VEGFR1 (Flt1), VEGFR2 (KDR/Flk1), and VEGFR3 (Flt4) [28]. In addition to VEGFA, the VEGF family of growth factors contains five other known members, namely placenta growth factor (PIGF), VEGFB, VEGFC, VEGF-D and viral VEGF homologs. Disruption of the genes encoding either VEGF or any of the three receptors of the VEGF family results in embryonic lethality due to failure of blood vessel development.

VEGFR2 is the main signal transducing VEGF receptor for angiogenesis and mitogenesis of endothelial cells. After receptor dimerization and autophosphorylation, several other signal transduction molecules are activated either directly or by indirect mechanisms [29].

VEGFA and MAPK were both up-regulated in RPE cells treated with either bevacizumab or ranibizumab. A differential involvement of mitogen activated protein kinases (MAPK) has been previously shown to be involved in VEGF expression and regulation [22], as P 38 is involved in constitutive and oxidative stress regulated VEGF expression. This pathway conveys the VEGF signal to microfilaments inducing rearrangements of the actin cytoskeleton that regulate endothelial cell migration by modulating the activation of MAPK2/3 (MAP kinase activated protein kinase-2/3) and phosphorylation of the F-actin polymerization modulator, HSP27 (heat shock protein-27).

KDR and NOS genes were both down-regulated in RPE cells exposed to both anti-VEGF agents. Both genes have key roles in mediating VEGF activity in increasing the proliferation and permeability of capillary endothelial cells.

Increasing the proliferation and permeability of endothelial cells may produce unwanted side effects, such as tumor angiogenesis, vascular leakage, edema, and inflammation [30]. In endothelial cells, the VEGF-Flk1/KDR signal system is a very important generator of NO (nitric oxide) through the activation of its downstream effectors PI3K, Akt kinase and eNOS (endothelial NO synthase).

Their role in RPE cells has also been previously investigated. KDR has been proposed to trasmit protective signaling against oxidant induced cell death in both normal conditions and disease states such as AMD [31]. It was also suggested to be involved in autokrine VEGF regulation [32].

Both genes were statistically significantly down-regulated after treatment with both anti-VEGF agents in RPE cell, perhaps indicating that both anti-VEGF agents block the angiogenesis and vascular leakage through these two genes.

Interestingly, both KDR and NOS3 genes were up-regulated in endothelial cells treated with bevacizumab compared to ranibizumab. This finding may suggest that there is a somewhat different mechanism of action between the two compounds in endothelial cells specifically.

Early preclinical [33, 34] and clinical [35–37] studies have implicated VEGF as a major factor in the pathogenesis of neovascular eye diseases, supporting the rationale for further investigation of the therapeutic potential of VEGF-targeted agents. VEGF-based therapies for a variety of human pathological situations that are associated with aberrant endothelial proliferation and aberrant neovascularization include the use of neutralizing antibodies against VEGFA or VEGFR2, antisense oligonucleotides, negative regulatory peptides, soluble receptors, and ATP analogs to inhibit the kinase activity of VEGFR [17]. Interference with VEGF function has, therefore, become a subject of major interest for drug development to block angiogenesis, and the targeting of the VEGF signaling pathway may be of therapeutic importance for many diseases.

## Conclusions

The present study describes the preliminary results of altered gene expression downstream of the VEGF signal pathway when comparing ranibizumab treatment to bevacizumab treatment in both RPE and endothelial cell lines. Our results indicate a somewhat different biological activity of the two compounds that need to be further explored.

This study has several limitation; first of being only based on RNA analysis (no Confirmation of results at the protein level) and results at the RNA level are often not replicated at the protein level.

The second limitation is that the study relies on a commercial custom-based Q-PCR assay, and the test genes were all genes in the VEGF pathway that were present on the kit. It could be that other more complex involvement of genes expression is involved and was not investigated.

The third limitation is the control groups chosen; because we have examined AMD model, and because of the complexity of the analysis of gene array, we have chosen not to further complex our statistics by adding non starved and non hypoxic cells.

Another major limitation of the study is the non conventional use of statistical analysis. We were unable to perform column analysis like t-testing or one way ANOVA because of the small number of samples obtained and the lack of normal distribution. In further studies, our results must be validated when using a larger scale analysis.
